# Factors Associated with Perinatal Depression and Anxiety Among Pregnant and Postpartum Women: A Cross-Sectional Study Based on Questionnaire Data

**DOI:** 10.3390/diseases14020067

**Published:** 2026-02-11

**Authors:** Byung Soo Kang, Jisoo Um, Subeen Hong, Hae-Jung Park, Joo Hyun Park, Jihyun Hwang, Tae-Suk Kim, Hyun Sun Ko

**Affiliations:** 1 Department of Obstetrics and Gynecology, Seoul St. Mary’s Hospital, College of Medicine, The Catholic University of Korea, Seoul 06591, Republic of Korea; 2 Department of Rehabilitation Medicine, Seoul St. Mary’s Hospital, College of Medicine, The Catholic University of Korea, Seoul 06591, Republic of Korea; 3 Department of Psychiatry, Seoul St. Mary’s Hospital, College of Medicine, The Catholic University of Korea, Seoul 06591, Republic of Korea

**Keywords:** perinatal depression, perinatal anxiety, mental health

## Abstract

**Background/Objectives**: Perinatal depression and anxiety are significant mental health concerns, and pharmacological treatments often pose considerable challenges. Therefore, this study aimed to evaluate the mental health status of pregnant and postpartum women and identify the factors affecting perinatal depression and anxiety. **Methods**: This cross-sectional study included 286 pregnant and postpartum women who completed questionnaires, including the Patient Health Questionnaire-9 (PHQ-9), Korean Version of the Edinburgh Postnatal Depression Scale (K-EPDS), and Generalized Anxiety Disorder-7 (GAD-7). **Results**: Symptoms of depression and anxiety were prevalent among participants. PHQ-9-positive cases were significantly less frequent in women from nuclear families, and their Pregnancy Stress Scale scores were significantly higher. K-EPDS-positive women had significantly lower rates of wanted pregnancies and marital satisfaction. GAD-7-positive cases showed significantly lower rates of wanted pregnancies, lower levels of social support, and higher Pregnancy Stress Scale scores. **Conclusions**: These findings highlight key psychosocial factors associated with perinatal depression and anxiety among pregnant and postpartum women, underscoring the importance of comprehensive mental health assessment during the perinatal period.

## 1. Introduction

Physical, psychological, and hormonal changes during pregnancy and postpartum make women vulnerable to depression and anxiety, which can affect their lifelong mental health. Several studies have reported that perinatal depression and anxiety in mothers are adversely associated with the poor development of social–emotional, cognitive, language, motor, and adaptive behaviors in their offspring in infancy, childhood, and beyond adolescence [1,2].

The global prevalence of perinatal depression is approximately 17.7%, ranging from 3% to 37% [3,4], while the prevalence of perinatal anxiety is 15–20% in women; these prevalences are higher in low- and middle-income countries [5,6,7]. In a nationwide study in Korea, 14.2% of participants had prenatal depression, and 24.3% had postpartum depression [8].

Untreated depression is associated with preterm birth, small for gestational age, stillbirth, low birth weight, and maternal morbidity [9,10]. Although it remains unclear whether antidepressant medication use during pregnancy is associated with an increased risk of adverse fetal and neonatal outcomes, such as congenital malformation, preterm birth, and neurodevelopmental problems in children [11,12,13,14,15], pregnancy is a determinant of antidepressant treatment modifications, especially treatment discontinuation. In a nationwide study in South Korea, 95.1% of women discontinued antidepressants during pregnancy [16]. However, the re-initiation rate of antidepressants is approximately 40% during pregnancy and postpartum [16,17,18].

Previous treatments for depression have been implemented through various methods, demonstrating that approaches besides pharmacological treatments can effectively improve maternal depression [19,20,21]. These therapeutic methods are based on the principle that the thoughts of an individual affect emotions and behaviors; behaviors, in turn, affect emotions and thoughts. However, these alternative therapies have limitations, including frequent clinic visits and lengthy counseling sessions. In the Korean society, social stigma, prejudice, and limited access to mental health services create significant barriers to the initiation of psychiatric treatment [22]. For pregnant or postpartum women, the physical discomfort of pregnancy and the challenges of childcare make regular and sustained visits to clinics particularly difficult. A recent treatment approach proposed to address these challenges involves the use of digital therapeutic platforms. Various methods have been attempted, and some have been developed; however, no approach has been commercialized for practical use in pregnant or postpartum women [23].

Because pregnant and postpartum women experience unique physical and psychological conditions distinct from those of the general population, personalized treatment approaches are essential. However, much of the existing research in this field relies heavily on subjective self-reports, which limits the ability to comprehensively and objectively evaluate perinatal mental health and its associated factors. To overcome these limitations, the use of standardized and validated measurement tools is necessary to ensure a more accurate and systematic assessment.

Accordingly, this study employed a range of established and validated instruments to evaluate mental health status and related psychosocial factors among pregnant and postpartum women. For postpartum participants, obstetric characteristics, including postpartum course, mode of delivery, gestational age at delivery, and infant birth weight, were additionally collected. Depressive and anxiety symptoms were assessed using the Patient Health Questionnaire-9 (PHQ-9), the Korean version of the Edinburgh Postnatal Depression Scale (K-EPDS), and the Generalized Anxiety Disorder-7 (GAD-7). The EPDS is the most widely used screening tool for depression during the perinatal and postpartum periods and is considered the gold standard for detecting postpartum depression. Its Korean version has been validated in multiple studies, with higher scores indicating more severe depressive symptoms [24,25,26]. The PHQ-9, developed for the detection of depression in primary care settings [27,28,29], and the GAD-7, a brief and reliable tool for assessing anxiety, have both been validated in Korean populations and are widely used in clinical and research settings [30].

In addition to mental health screening, this study assessed a broad range of psychosocial and contextual factors related to life satisfaction. These included marital satisfaction [31], division of household and childcare responsibilities, perceived support from individuals other than the spousal support [24], Multidimensional Scale of Perceived Social Support (MSPSS) [25], and satisfaction with the parental role [26]. To capture pregnancy-related physical and psychological experiences, participants were also asked about discomfort associated with physical changes during pregnancy, self-perception of body changes, pregnancy-related stress factors (including concerns about maternal and fetal health, maternal role, and social environment), and coping strategies for anxiety. These measures were adapted from previously developed and validated scales and applied in a manner appropriate to the Korean cultural context.

Furthermore, recognizing the growing interest in digital approaches to mental health care, this study exploratorily examined participants’ subjective opinions regarding a perinatal mental health digital therapeutic platform. Specifically, participants were asked about their level of agreement with the development of digital therapeutics and their willingness to use such a platform, along with the reasons for their responses. This component was included to provide preliminary insight into perceptions of emerging therapeutic modalities rather than to propose or evaluate a specific intervention.

Through the use of standardized measurement tools and a multidimensional assessment framework, this study aimed to provide a more objective and comprehensive evaluation of mental health status and associated factors among pregnant and postpartum women.

## 2. Methods

### 2.1. Study Design

This study was designed as a cross-sectional study to assess the mental health status of pregnant and postpartum women and to identify factors associated with perinatal depression and anxiety. Data were collected over a defined period, from 23 February 2023 to 16 May 2024. Each participant completed the survey once, and no follow-up assessment was conducted.

### 2.2. Participants

The study included women who were pregnant or within 1 year postpartum (aged 19–49 years) and who voluntarily participated. The survey was conducted online, and participants were recruited by distributing information leaflets to women visiting or admitted to the outpatient clinic at Seoul St. Mary’s Hospital. The leaflets contained a QR code that the women voluntarily used to access and complete the online survey. Detailed information regarding the study purpose, participation procedures, and data handling was provided on the introductory page of the online survey and is also included in Supplementary Material S2. The survey was administered online and comprised 231 items. Participants who completed the survey received a small coupon as compensation. Responses from incomplete surveys were not collected.

### 2.3. Sample Size Calculation

The sample size was calculated based on the estimated number of deliveries in South Korea. According to national statistics, the total number of live births in 2021 was approximately 260,500 [31]. Based on these figures, the estimated number of deliveries over six months was approximately 125,000. Using a 95% confidence level, a 5% margin of error, and a confidence interval width of 10%, the required sample size was calculated. Based on the assumption that approximately 90% of eligible women visiting the outpatient clinic at Seoul St. Mary’s Hospital would be able to understand the study explanation and meet the eligibility criteria, a minimum of 139 participants was required. Considering an anticipated survey response rate of approximately 50% (corresponding to an estimated dropout or non-completion rate of 50%), the target sample size was increased to 278 participants. The detailed calculation method is provided in Supplementary Material S1.

### 2.4. Survey Questionnaires

Sociodemographic characteristics: age, height, weight, pre-pregnancy weight, marital status, family structure, age of spouse, duration of marriage (or cohabitation), place of residence, occupation, educational level, and personal and family income.

Clinical characteristics: pregnancy status, gestational age or postpartum months, pregnancy method and planning status, gravidity and parity, previous experience of depression during or after pregnancy, preexisting conditions, pregnancy complications, smoking/alcohol history, and physical activity (International Physical Activity Questionnaire [IPAQ]) [27,28]. Spearman Rho coefficients and Kappa values of test–retest reliability were 0.427~0.646 (median 0.542) and 0.365~0.620 (median 0.471).

Postpartum women: postpartum course, delivery method, gestational age at delivery, birth weight.

Depression/anxiety screening tools: depression and anxiety were measured using the Patient Health Questionnaire-9 (PHQ-9), the Korean Version of the EPDS (K-EPDS), and the Generalized Anxiety Disorder-7 (GAD-7).

The most commonly used tool for assessing depression during the perinatal and postpartum periods is the EPDS; the Korean version (K-EPDS) was utilized in this study. The EPDS is widely used and considered the gold standard for detecting postpartum depression [29]. Its validity has been confirmed in multiple studies in Korea (Cronbach’s alpha = 0.85) [29], with a score of 9 or higher indicating moderate or severe depressive symptoms [32,33]. The PHQ-9 was developed based on the Primary Care Evaluation of Mental Disorders Procedure for the detection of depression in primary care settings. Its validity has been established, with a score of 10 or higher typically indicating moderate or severe depressive symptoms [34,35,36]. In addition, the Korean version of the PHQ-9 has been formally validated in Korean populations and has demonstrated good reliability and construct validity across clinical and community-based samples (Cronbach’s alpha = 0.81~0.85) [37,38]. The GAD-7 is a simple tool for diagnosing anxiety, with a score of 5 or higher indicating moderate or severe anxiety. It has been validated in Korea and widely used in various contexts [39]. It has been validated in Korean populations and is widely used across various clinical and research settings, demonstrating excellent internal consistency (Cronbach’s α = 0.93) [37,40].

### 2.5. Data Analysis

The collected data were analyzed using the SPSS Statistics software (version 27.0; IBM Corp., Armonk, NY, USA). For the depression/anxiety screening tools, moderate or high levels of discomfort were defined as PHQ-9 scores ≥ 10, K-EPDS scores ≥ 9, and GAD-7 scores ≥ 5.

The internal consistency of the PHQ-9, K-EPDS, and GAD-7 in the current sample was evaluated using Cronbach’s alpha coefficients. Group differences in sociodemographic and clinical characteristics according to screening status were examined using the chi-square test (or Fisher’s exact test when expected cell counts were <5) for categorical variables and the independent samples *t*-test (or the Mann–Whitney U test for non-normally distributed variables) for continuous variables.

Multivariable logistic regression analyses were conducted to identify factors independently associated with depression and anxiety. Variables included in the multivariable models were selected based on clinical relevance and statistical significance in univariable analyses. Prior to model construction, conceptually overlapping psychosocial variables were carefully reviewed, and representative variables were selected to improve model stability and interpretability. In addition, the assumptions for logistic regression were assessed, and multicollinearity among independent variables was evaluated using variance inflation factors (VIFs); all VIF values were below the commonly accepted threshold of 10. Statistical significance was set at *p* < 0.05.

For the multivariable logistic regression analyses, the dependent variables were binary indicators of depression and anxiety based on screening tool cut-off scores. Specifically, PHQ-9 positivity (PHQ-9 ≥ 10), K-EPDS positivity (K-EPDS ≥ 9), and GAD-7 positivity (GAD-7 ≥ 5) were each used as dependent variables in separate regression models. All selected independent variables were entered simultaneously into the multivariable logistic regression models. Adjusted odds ratios (ORs) with 95% confidence intervals (CIs) were calculated to estimate the strength of associations.

### 2.6. Ethical Considerations

This study was approved by the Institutional Review Board (IRB) of Seoul St. Mary’s Hospital prior to commencement to ensure the protection of the study participants (IRB approval number: KC22QNSI0786). All participants voluntarily participated in the study after being fully informed of the study’s purpose, and all personal information was processed anonymously. The collected data will be stored for 3 years after the conclusion of the study and destroyed afterward.

## 3. Results

### 3.1. Demographic Characteristic

In total, 286 responses were obtained. Among them, 45 participants screened positive for depression using the PHQ-9, 73 screened positive using the K-EPDS, and 63 screened positive for anxiety using the GAD-7.

The sociodemographic and clinical characteristics of the study participants are summarized in Table 1. Comparisons of characteristics according to screening status for each assessment tool are presented in Table 2. Participants from large families showed a significantly higher rate of positive screening on the PHQ-9 (*p* = 0.01). Meanwhile, participants with unwanted pregnancies had significantly higher positive screening rates on the K-EPDS and GAD-7 (*p* = 0.02 and *p* = 0.006, respectively).

The internal consistency of the screening instruments was good to excellent, with Cronbach’s alpha coefficients of 0.86 for the PHQ-9, 0.89 for the K-EPDS, and 0.90 for the GAD-7 (Supplementary Material S4).

### 3.2. Life Satisfaction

Table 3 shows that the Life Satisfaction Scale yielded similar results across all three screening tools. Participants with lower job satisfaction had significantly higher positive screening rates (PHQ-9 *p* = 0.046, K-EPDS *p* = 0.002, GAD-7 *p* = 0.029). In addition, lower marital satisfaction was significantly associated with higher positive screening rates (PHQ-9 *p* = 0.003, K-EPDS *p* < 0.001, GAD-7 *p* < 0.001). Additionally, lower scores on the Spousal Support Scale (PHQ-9 *p* = 0.005, K-EPDS *p* < 0.001, GAD-7 *p* < 0.001) and Social Support Scale (PHQ-9 *p* < 0.001, K-EPDS *p* < 0.001, GAD-7 *p* < 0.001) were significantly associated with positive screening results. Parental role satisfaction was significantly lower in the positive screening group (PHQ-9 *p* < 0.001, K-EPDS *p* < 0.001, GAD-7 *p* < 0.001). Self-perception regarding body changes was significantly lower in the positive screening group (PHQ-9 *p* = 0.018, K-EPDS *p* < 0.001, GAD-7 *p* = 0.001), whereas thoughts on stress factors during pregnancy were significantly higher in this group (PHQ-9 *p* < 0.001, K-EPDS *p* < 0.001, GAD-7 *p* < 0.001). Positive coping strategies for anxiety were significantly associated with lower positive screening rates (PHQ-9 *p* = 0.006, K-EPDS *p* = 0.002, GAD-7 *p* = 0.019). 

### 3.3. Degree of Discomfort with Physical Changes During Pregnancy

Table 4 shows that participants who reported hair loss (*p* = 0.027), edema (*p* = 0.02), and changes in appetite (*p* = 0.03) had significantly higher positive screening rates in the PHQ-9. Hair loss (*p* = 0.001), hemorrhoids (*p* = 0.013), changes in appetite (*p* < 0.001), and pain (*p* = 0.045) were significantly associated with positive screening results in the K-EPDS. For the GAD-7, weight gain (*p* = 0.029), hair loss (*p* = 0.001), hemorrhoids (*p* = 0.014), and changes in appetite (*p* = 0.003) were significantly associated with positive screening results.

### 3.4. Multivariate Regression Analysis

Table 5 presents the results of multivariate regression analysis for variables that were significant in the univariable analyses. For the PHQ-9, participants from nuclear families were significantly less likely to have positive screening results (*p* = 0.03, OR = 0.155); higher scores on thoughts about stress factors during pregnancy were significantly associated with higher positive screening rates on the PHQ-9 (*p* = 0.025, OR = 1.048).

Variables affecting the K-EPDS scores included whether the pregnancy was wanted and marital satisfaction, with a wanted pregnancy being associated with a lower likelihood of positive screening (*p* = 0.034, OR = 0.175); higher marital satisfaction was associated with lower positive screening rates (*p* = 0.013, OR = 0.231).

For the GAD-7, wanted pregnancy was associated with a lower likelihood of positive screening (*p* = 0.028, OR = 0.161), and an increase in the degree of social support was significantly associated with a lower likelihood of positive screening (*p* = 0.045, OR = 0.970). Finally, an increase in thoughts about stress factors during pregnancy was significantly associated with higher positive screening rates on the GAD-7 (*p* = 0.014, OR = 1.047).

## 4. Discussion

In this study, multivariable logistic regression analyses identified several psychosocial and pregnancy-related factors independently associated with perinatal depression and anxiety among pregnant and postpartum women. Across all three screening instruments, thoughts related to stress factors during pregnancy emerged as a consistent and significant correlate of both depressive and anxiety symptoms, underscoring the central role of pregnancy-related stress in perinatal mental health.

Using the PHQ-9, K-EPDS, and GAD-7 screening tools, the present study identified the characteristics of pregnant and postpartum women with moderate-to-severe depressive symptoms and anxiety. Our results showed that the PHQ-9-positive group was significantly less likely to belong to a nuclear family than to a large family and had significantly higher scores on the Pregnancy Stress Scale (PSS), indicating greater perceived stress during pregnancy. Women who screened positive on the K-EPDS were less likely to report wanted pregnancies and reported significantly lower marital satisfaction. Additionally, those who tested positive on the GAD-7 were less likely to have wanted pregnancies and reported lower levels of perceived social support. In addition, they had significantly higher PSS scores, indicating higher perceived stress during pregnancy.

Thoughts on stress factors during pregnancy, as assessed by the Pregnancy Stress Scale, reflect the perceived burden, worry, and difficulty associated with various aspects, such as “physical and psychological changes,” “coping in daily life,” “health of the mother and baby,” “maternal role,” “family support,” “healthcare services,” “social atmosphere,” and “reconciliation of work life.” It captures the issues perceived by women, irrespective of the actual situation [41]. Similarly, marital satisfaction refers to the degree of satisfaction that the mother perceives with her marriage. The present findings suggest that these perceptual and cognitive factors may play a particularly important role in perinatal mental health, as pregnancy-related stress consistently showed significant associations with screening outcomes, whereas sociodemographic and obstetric variables demonstrated more limited effects. In this context, the concept of a “wanted pregnancy” may be interpreted as reflecting the mother’s perception and acceptance of pregnancy and childbirth, rather than a purely demographic characteristic.

Regarding the significantly lower PHQ-9 positivity in nuclear families compared to large families, it might initially seem that larger families could provide more help with child rearing and household chores. However, the opposite may be true because of potential conflicts with older generations, especially the traditionally known conflicts between mothers-in-law and daughters-in-law, which cannot be overlooked [42].

Previous studies in South Korea have suggested younger (<20 years) and advanced maternal age (≥35 years), primiparity, previous depression, peripartum hysterectomy, uterine artery embolization, preterm delivery, placental abruption, cesarean delivery, induced labor, and preeclampsia as obstetric risk factors for postpartum depression [8]. Another recent study on postpartum depression suggested that depressive feelings at 12 weeks of gestation and postpartum factors of stress, relationship with children, depressive feelings, fear, sadness, and neonatal intensive care unit admission were associated with a higher risk of postpartum depression. In contrast, high postpartum quality of life and marital satisfaction at postpartum were significantly associated with a lower risk of postpartum depression. Other longitudinal studies conducted to predict postpartum depression have identified maternal history, family history, and sociodemographic status as significant factors [43].

A key strength of this study is its effort to minimize reliance on subjective impressions by employing multiple validated and standardized screening instruments to quantitatively assess depressive and anxiety symptoms. The use of the PHQ-9, K-EPDS, and GAD-7 allowed symptoms to be measured in a structured and reproducible manner, enabling the identification of diverse symptom profiles and associated factors through statistical analysis. In addition to basic sociodemographic characteristics, the study incorporated a range of validated measures capturing mothers’ perceived stress, social support, and relational context, thereby facilitating a more comprehensive and objectively grounded evaluation of the perinatal environment. This emphasis on quantified assessment and validated measurement enhances the interpretability and reliability of the findings.

However, several limitations must be acknowledged. This study was conducted as a cross-sectional study, without accounting for the different mood changes that can occur at various stages of pregnancy. In addition, the characteristics of pregnant women quite differ from those of postpartum women; however, the study did not separate and analyze these groups, which is a notable limitation. Future studies should separately analyze data before and after childbirth to yield more meaningful results. Another limitation is the survey design and data collection method. The questionnaire consisted of 231 items and was administered online via a QR code. The length of the survey may have increased respondent burden and response fatigue, potentially discouraging participation or leading to incomplete responses. Consequently, women who were more motivated, had greater interest in mental health, or had sufficient time and digital literacy may have been more likely to complete the survey. In addition, because participation relied on voluntary access through a QR code, the study population may not be fully representative of the broader population of pregnant and postpartum women. These factors may have introduced selection bias and limited the generalizability of the findings. Furthermore, depression and anxiety were assessed using self-reported screening tools rather than clinical diagnostic interviews. Although these instruments are validated for identifying individuals at risk, they do not constitute definitive diagnoses. Therefore, the findings should be interpreted as screening-positive symptoms.

Importantly, the relatively small sample size represents a key methodological limitation. Due to inadequate statistical power, it was not possible to include all theoretically relevant variables in the multivariate models, and robust multivariate adjustment could not be fully performed. As a result, some potentially important confounding factors may not have been adequately controlled, and the findings should be interpreted with caution. Future studies with larger sample sizes and prospective power calculations are needed to enable more comprehensive multivariate analyses and to strengthen the validity and generalizability of the results. Although multicollinearity was assessed and addressed, some conceptual overlap among psychosocial variables may remain.

Despite these limitations, this study provides meaningful insight into the psychosocial characteristics associated with depressive and anxiety symptoms among pregnant and postpartum women. The findings underscore the importance of comprehensive psychosocial assessment during the perinatal period and may inform the development of future non-pharmacological approaches tailored to this population.

## 5. Conclusions

This study demonstrated that depressive and anxiety symptoms among pregnant and postpartum women are associated with psychosocial and relational factors, including marital satisfaction, perceived social support, family structure, and pregnancy intention. These findings highlight the importance of a comprehensive clinical assessment that integrally reflects women’s subjective experiences and relational contexts. Routine mental health screening during the perinatal period, together with early, integrated non-pharmacological interventions focusing on stress management and enhancement of relational and social support, may play a critical role in improving maternal mental health outcomes.

## Figures and Tables

**Table 1:** Demographic and Clinical Characteristics of the Study Participants.

Variable	*N*	Value	Min–Max
Maternal age (years)	286	34.04 ± 3.72	19–42
Pregnant women (*N* = 210)			
Gestational age of current pregnancy (weeks)	210	25.33 ± 8.29	5–39
Postpartum women (*N* = 76)			
Postpartum days	76	23.51 ± 38.16	0–240
Gestational age at delivery (weeks)	76	36.91 ± 4.14	10–40
Delivery type (*N* = 76)			
Cesarean section	46	46 (60.5%)	
Vaginal delivery	30	30 (39.5%)	
Birth weight of newborn (kg)	76	2.96 ± 0.57	1.0–4.0
Paternal age (years)	286	36.14 ± 4.48	20–51
Duration of marriage/cohabitation (years)	284	3.27 ± 2.53	1–10
Height (cm)	286	162.03 ± 4.83	145–177
Weight, current (kg)	286	64.14 ± 9.53	46–104
Preexisting underlying diseases before pregnancy (*N* = 286)			
Yes	51	51 (17.8%)	
No	235	235 (82.2%)	
Diseases diagnosed during pregnancy			
Yes	78	78 (27.3%)	
No	208	208 (72.7%)	

Values are presented as mean ± standard deviation or number (%), as appropriate.

**Table 2:** Comparison of sociodemographic and clinical characteristics according to screening results.

	PHQ-9			K-EPDS			GAD-7		
	No	Yes	*p*	No	Yes	*p*	No	Yes	*p*
	(*N* = 241)	(*N* = 45)		(*N* = 213)	(*N* = 73)		(*N* = 223)	(*N* = 63)	
Age	33.0 [32.0; 36.0]	35.0 [32.0; 37.0]	0.248	33.0 [31.0; 36.0]	34.0 [32.0; 36.0]	0.236	33.0 [32.0; 36.0]	34.0 [32.0; 36.0]	0.614
pregnancy status			0.842			0.519			
-yes	178 (73.9%)	32 (71.1%)		159 (74.6%)	51 (69.9%)		166 (74.4%)	44 (69.8%)	
-no	63 (26.1%)	13 (28.9%)		54 (25.4%)	22 (30.1%)		57 (25.6%)	19 (30.2%)	
Gestational age (if pregnant) (weeks)	24.0 [20.0; 33.0]	26.0 [19.5; 33.0]	0.913	24.0 [20.0; 33.0]	26.0 [20.5; 32.5]	0.898	24.0 [20.0; 32.0]	26.0 [19.5; 33.0]	0.988
postpartum period (days)	12.0 [3.0; 21.0]	14.0 [6.0; 42.0]	0.505	14.0 [7.0; 21.0]	8.5 [2.0; 42.0]	0.522	11.0 [3.0; 14.0]	14.0 [6.5; 45.5]	0.188
Methods of achieving pregnancy			0.194			0.367			0.489
-COH	5 (2.1%)	3 (6.7%)		5 (2.3%)	3 (4.1%)		5 (2.2%)	3 (4.8%)	
-IVF-ET	41 (17.0%)	11 (24.4%)		38 (17.8%)	14 (19.2%)		43 (19.3%)	9 (14.3%)	
-IVF-IUI	12 (5.0%)	2 (4.4%)		13 (6.1%)	1 (1.4%)		12 (5.4%)	2 (3.2%)	
-Natural pregnancy	183 (75.9%)	29 (64.4%)		157 (73.7%)	55 (75.3%)		163 (73.1%)	49 (77.8%)	
Income (~10,000 won) (personal)	350.0 [50.0; 350.0]	350.0 [150.0; 450.0]	0.341	350.0 [50.0; 450.0]	350.0 [50.0; 450.0]	0.985	350.0 [50.0; 350.0]	350.0 [200.0; 450.0]	0.447
wanted pregnancy			0.192			0.02			0.006
-unwanted	8 (3.3%)	4 (8.9%)		5 (2.3%)	7 (9.6%)		5 (2.2%)	7 (11.1%)	
-wanted	233 (96.7%)	41 (91.1%)		208 (97.7%)	66 (90.4%)		218 (97.8%)	56 (88.9%)	
Family type			0.01			0.15			0.314
-large family	5 (2.1%)	5 (11.1%)		5 (2.3%)	5 (6.8%)		6 (2.7%)	4 (6.3%)	
-nuclear family	236 (97.9%)	40 (88.9%)		208 (97.7%)	68 (93.2%)		217 (97.3%)	59 (93.7%)	
Height (cm)	162.0 ± 4.8	162.3 ± 5.0	0.657	161.8 ± 4.7	162.8 ± 5.2	0.133	162.1 ± 4.8	161.9 ± 5.1	0.742
Body weight (kg)	63.0 [57.0; 70.0]	63.0 [57.0; 69.0]	0.716	62.0 [57.0; 70.0]	64.0 [59.8; 70.0]	0.219	63.0 [57.4; 70.0]	63.0 [56.4; 70.0]	0.794
Body weight before pregnancy (kg)	56.0 [51.5; 61.5]	56.0 [52.0; 60.0]	0.863	55.0 [51.0; 61.0]	57.0 [52.0; 61.0]	0.429	55.5 [52.0; 61.0]	56.0 [52.0; 60.0]	0.59
BMI > 30 (before pregnancy)			0.667			0.234			0.347
-no	228 (95.0%)	44 (97.8%)		200 (94.3%)	72 (98.6%)		210 (94.6%)	62 (98.4%)	
-yes	12 (5.0%)	1 (2.2%)		12 (5.7%)	1 (1.4%)		12 (5.4%)	1 (1.6%)	
smoking			0.053			0.087			0.215
-no	236 (97.9%)	41 (91.1%)		209 (98.1%)	68 (93.2%)		218 (97.8%)	59 (93.7%)	
-yes	5 (2.1%)	4 (8.9%)		4 (1.9%)	5 (6.8%)		5 (2.2%)	4 (6.3%)	
drinking			0.718			1			0.07
-no	240 (99.6%)	44 (97.8%)		212 (99.5%)	72 (98.6%)		223 (100.0%)	61 (96.8%)	
-yes	1 (0.4%)	1 (2.2%)		1 (0.5%)	1 (1.4%)		0 (0.0%)	2 (3.2%)	
Intense physical exercise			0.195			0.217			0.154
-no	213 (88.4%)	36 (80.0%)		189 (88.7%)	60 (82.2%)		198 (88.8%)	51 (81.0%)	
-yes	28 (11.6%)	9 (20.0%)		24 (11.3%)	13 (17.8%)		25 (11.2%)	12 (19.0%)	
Light physical exercise			0.472			0.483			1
-no	106 (44.0%)	23 (51.1%)		93 (43.7%)	36 (49.3%)		101 (45.3%)	28 (44.4%)	
-yes	135 (56.0%)	22 (48.9%)		120 (56.3%)	37 (50.7%)		122 (54.7%)	35 (55.6%)	
Underlying disease			0.14			0.052			0.112
-no	202 (83.8%)	33 (73.3%)		181 (85.0%)	54 (74.0%)		188 (84.3%)	47 (74.6%)	
-yes	39 (16.2%)	12 (26.7%)		32 (15.0%)	19 (26.0%)		35 (15.7%)	16 (25.4%)	
Diseases diagnosed during pregnancy			0.239			0.628			0.288
-no	179 (74.3%)	29 (64.4%)		157 (73.7%)	51 (69.9%)		166 (74.4%)	42 (66.7%)	
-yes	62 (25.7%)	16 (35.6%)		56 (26.3%)	22 (30.1%)		57 (25.6%)	21 (33.3%)	

Continuous variables are presented as median with interquartile ranges (25th–75th percentiles), and categorical variables are presented as numbers (%). *p*-values were calculated using the chi-square test or Fisher’s exact test for categorical variables and the independent samples *t*-test or Mann–Whitney U test for continuous variables, as appropriate.

**Table 3:** Life Satisfaction.

	PHQ-9			K-EPDS			GAD-7		
	Job satisfaction								
	31.0 [27.0; 37.0]	28.0 [27.0; 35.0]	0.046	32.4 [25.7; 39.1]	29.3 [23.7; 35.0]	0.002	32.0 [27.0; 37.0]	28.0 [26.0; 35.0]	0.029
	Marital satisfaction								
			0.003			0			<0.001
-satisfied	130 (53.9%)	13 (28.9%)		123 (57.7%)	20 (27.4%)		125 (56.1%)	18 (28.6%)	
-not satisfied	111 (46.1%)	32 (71.1%)		90 (42.3%)	53 (72.6%)		98 (43.9%)	45 (71.4%)	
	Spousal Support Scale								
	15.0 [12.0; 18.0]	13.0 [11.0; 16.0]	0.005	16.0 [13.0; 18.0]	12.0 [11.0; 15.0]	0	15.0 [13.0; 18.0]	13.0 [11.0; 15.5]	<0.001
	Level of Husband’s Involvement in Childcare and Housework (50% or More)								
			1			0.709			1
-no	103 (42.7%)	19 (42.2%)		89 (41.8%)	33 (45.2%)		95 (42.6%)	27 (42.9%)	
-yes	138 (57.3%)	26 (57.8%)		124 (58.2%)	40 (54.8%)		128 (57.4%)	36 (57.1%)	
	Social Support Scale								
	58.0 [47.0; 67.0]	46.0 [35.0; 58.0]	<0.001	58.0 [48.0; 67.0]	48.0 [36.0; 59.0]	<0.001	58.0 [48.0; 67.0]	44.0 [34.0; 57.5]	<0.001
	Level of Involvement from Others (excluding Husband) in Childcare and Housework (50% or More)								
			0.735			1			0.815
-no	162 (67.2%)	32 (71.1%)		144 (67.6%)	50 (68.5%)		150 (67.3%)	44 (69.8%)	
-yes	79 (32.8%)	13 (28.9%)		69 (32.4%)	23 (31.5%)		73 (32.7%)	19 (30.2%)	
	Parental role satisfaction								
	52.0 [46.0; 59.0]	47.0 [39.0; 52.0]	<0.001	53.0 [47.0; 60.0]	47.0 [38.0; 52.0]	<0.001	52.0 [47.0; 60.0]	47.0 [38.0; 54.0]	<0.001
	Self-perception regarding body changes								
	30.0 [23.0; 38.0]	24.0 [19.0; 33.0]	0.018	30.0 [23.0; 38.0]	24.0 [16.0; 33.0]	<0.001	30.0 [23.0; 38.0]	24.0 [19.0; 31.5]	0.001
	Thoughts on stress factors during pregnancy								
	34.2 ± 11.3	41.0 ± 12.9	<0.001	33.6 ± 11.5	40.2 ± 11.4	<0.001	33.9 ± 11.6	39.9 ± 11.5	<0.001
	Coping strategies for anxiety								
			0.006			0.002			0.019
-no	61 (25.3%)	21 (46.7%)		50 (23.5%)	32 (43.8%)		56 (25.1%)	26 (41.3%)	
-yes	180 (74.7%)	24 (53.3%)		163 (76.5%)	41 (56.2%)		167 (74.9%)	37 (58.7%)	

**Table 4:** Degree of discomfort with physical changes during pregnancy (*p*-value).

	PHQ-9	K-EPDS	GAD-7
fatigue	0.597	0.192	0.088
dizziness	0.215	0.05	0.079
weight gain	0.294	0.166	0.029
hair loss	0.027	0.001	0.001
edema	0.02	0.152	0.103
skin change	0.75	0.849	0.124
hyperemesis gravidarum	0.098	0.647	0.158
constipation	1	0.499	0.337
hemorrhoids	0.071	0.013	0.014
change appetite	0.03	0	0.003
bleeding	0.6	0.09	0.034
vaginal discharge	0.753	0.126	0.598
pain	0.617	0.045	0.087
Urinary symptom	0.284	0.109	0.164

**Table 5:** Multivariate regression analysis for the variables that were significant in the previous analyses.

**PHQ-9**								
Model	B	S.E.	Wald	df	*p*-value	OR	95% CI for OR	
							Lower	Upper
Family structure-nuclear family	−1.866	0.859	4.719	1	0.030	0.155	0.029	0.833
Thoughts on stress factors during pregnancy	0.047	0.021	5.024	1	0.025	1.048	1.006	1.093
Coping strategies for anxiety	−0.724	0.415	3.044	1	0.081	0.485	0.215	1.093
**K-EPDS**								
Model	B	S.E.	Wald	df	*p*-value	OR	95% CI for OR	
							Lower	Upper
wanted pregnancy	−1.745	0.825	4.477	1	0.034	0.175	0.035	0.879
Marital satisfaction	−1.466	0.589	6.199	1	0.013	0.231	0.073	0.732
Thoughts on stress factors during pregnancy	0.041	0.019	4.621	1	0.032	1.042	1.004	1.083
**GAD-7**								
Model	B	S.E.	Wald	df	*p*-value	OR	95% CI for OR	
							Lower	Upper
wanted pregnancy	−1.825	0.831	4.818	1	0.028	0.161	0.032	0.822
Support from the surroundings	−0.030	0.015	4.026	1	0.045	0.970	0.942	0.999
Thoughts on stress factors during pregnancy	0.046	0.019	6.093	1	0.014	1.047	1.010	1.087

## Data Availability

The data supporting the findings of this study are not publicly available due to privacy and ethical restrictions involving human participants, but are available from the corresponding author upon reasonable request.

## Supplementary Materials

The following supporting information can be downloaded at https://www.mdpi.com/article/10.3390/diseases14020067/s1. Supplementary Material S1: Sample size calculation; Supplementary Material S2: Participant information sheet; Supplementary Material S3: Questionnaire; Supplementary Material S4: Cronbach’s alpha results.
