# Supplementary material for: Factors Associated with Perinatal Depression and Anxiety Among Pregnant and Postpartum Women: A Cross-Sectional Study Based on Questionnaire Data
Source: Diseases. 2026 Feb 11;14(2):67. doi: 10.3390/diseases14020067 (PMC12939594; doi:10.3390/diseases14020067)
Supplement: Supplementary file 1 [file diseases-14-00067-s001.zip › Supplementary Material S1. Sample size calculation.pdf]

[calculation program: PASS 16]

Confidence Intervals for One Proportion from a Finite Population

Numeric Results

Confidence Level	Sample Size (n)	Precision or Half-Width (d)	Sample Proportion (P)	Population Size (N)
0.950	139	0.0500	0.9000	125000

Dropout-Inflated Sample Size

Dropout Rate	Sample Size n	Dropout-Inflated Enrollment Sample Size n'	Expected Number of Dropouts D
50%	139	278	139
