# Supplementary material for: Factors Associated with Perinatal Depression and Anxiety Among Pregnant and Postpartum Women: A Cross-Sectional Study Based on Questionnaire Data
Source: Diseases. 2026 Feb 11;14(2):67. doi: 10.3390/diseases14020067 (PMC12939594; doi:10.3390/diseases14020067)
Supplement: Supplementary file 1 [file diseases-14-00067-s001.zip › Supplementary Material S2. The participant information sheet.pdf]

Participant Information Sheet for Survey Study

Hello,

We are a research team from the Departments of Obstetrics and Gynecology, Psychiatry, and Rehabilitation Medicine at Seoul St. Mary's Hospital, College of Medicine, The Catholic University of Korea. Our team is currently conducting a study titled:

"Development of a Digital Treatment Device Platform for Perinatal Maternal Mental Health"

supported by the Korea Health Industry Development Institute (KHIDI).

In order to develop an effective digital treatment platform for perinatal maternal mental health, we believe that the opinions and experiences of pregnant women or women who have recently given birth are essential. Therefore, we are conducting this survey.

The survey will take approximately 10 minutes, and participants who complete the survey will receive a small token of appreciation.

Confidentiality and Voluntary Participation

- This survey is conducted anonymously.
- All responses will be kept strictly confidential.
- Participation is completely voluntary, and you may discontinue participation at any time without any disadvantage or penalty.
- You may skip any questions you feel uncomfortable answering.

We sincerely ask for your honest and thoughtful responses, and we thank you for taking the time to participate.

You may access the online questionnaire via the link or QR code below:

Principal Investigator
Hyun Sun Ko, M.D., Ph.D.
Department of Obstetrics and Gynecology, Seoul St. Mary's Hospital
The Catholic University of Korea
Research Team for the Development of a Management Model for Gestational Diabetes Mellitus

Contact Information

For inquiries regarding this study, please contact:

Dr. Hyun Sun Ko (Department of Obstetrics and Gynecology, Seoul St. Mary's Hospital)
Email: mongkoko@catholic.ac.kr

Seoul St. Mary's Hospital Institutional Review Board (IRB) Office:
+82-2-2258-8195~8201

Catholic Medical Center Central Institutional Review Board (Human Research Protection Unit):
+82-2-2258-8202~8206

Online Survey Link:

https://docs.google.com/forms/d/e/1FAIpQLSednlSZyjkjcnCS5V0T_nUYQPwwXflIbkxDWJM8hSWPgTvozA/viewform?usp=sf_link
