# Supplementary material for: Factors Associated with Perinatal Depression and Anxiety Among Pregnant and Postpartum Women: A Cross-Sectional Study Based on Questionnaire Data
Source: Diseases. 2026 Feb 11;14(2):67. doi: 10.3390/diseases14020067 (PMC12939594; doi:10.3390/diseases14020067)
Supplement: Supplementary file 1 [file diseases-14-00067-s001.zip › Supplementary Material S3. questionnare.pdf]

1. **What is your age?**
2. **Are you currently pregnant?**
3. **What is your current gestational age (weeks)?**
4. **How long has it been since you gave birth?**
5. **What was the mode of delivery?**
6. **At how many weeks of gestation did delivery occur?**
7. **What was the birth weight of the newborn?**
8. **What was the method of conception for this pregnancy?**
9. **Was this a planned pregnancy?**
10. **Have you had any previous childbirth experience?** (vaginal delivery or cesarean section at ≥20 weeks of gestation)
11. **If you have had previous childbirth experience, what was the year of your most recent delivery?** (_____ year)
12. **If applicable, how many childbirth experiences have you had?**
13. **Have you ever experienced preterm birth?** (delivery between ≥20 weeks and <37 weeks of gestation)
14. **Have you ever experienced depression during a previous pregnancy or postpartum period?**
15. **Have you ever experienced miscarriage (before 20 weeks) or stillbirth (after 20 weeks)?**
16. **How many fetuses are you currently carrying?**
17. **What is your current marital status?**
18. **What is the age of your spouse (the father of the baby)?** (____ years)
19. **What is your current family structure?**
20. **If currently married or cohabiting, how long have you been married or living together?**
21. **Have you been diagnosed with any medical conditions during this pregnancy?**
22. **Have you been diagnosed with any chronic medical conditions prior to pregnancy?**
23. **What is your height?** (____ cm)
24. **What is your current body weight?** (____ kg)
25. **What was your body weight before pregnancy?** (____ kg)
26. **What is your smoking status?**
27. **If you smoke, what was/is your average daily smoking amount?**
    Current: ____ packs (or cigarettes) per day
    Before pregnancy: ____ packs (or cigarettes) per day
28. **What is your alcohol consumption history?**
29. **If you consume alcohol, how often do you drink on average?**
30. **On drinking days, how many standard drinks do you usually consume at one time?**
31. **During the past 7 days, did you engage in vigorous physical activities that made you breathe much harder than usual?**
32. **If yes, how many days per week and for how long each day?**
    (____ days per week, ____ hours per day)
33. **During the past 7 days, did you engage in light physical activities such as brisk walking for at least 10 minutes?**
34. **If yes, how many days per week and for how long each day?**
    (____ days per week, ____ hours per day)
35. **During the past 7 days, how much time did you usually spend sitting on a weekday?**
    ____ hours ____ minutes per day
36. **What is your current area of residence?**

37. **What is your current occupation?**
38. **Please indicate the response that best reflects your opinion.**
    A. I am satisfied with my current income. (Yes / No)
    B. My current job is stable.
    C. I am satisfied with the content of my current work.
    D. I am satisfied with my working environment.
    E. I am satisfied with my working hours.
    F. There are many opportunities for personal development at my current workplace.
    G. I am satisfied with communication and relationships with my colleagues.
    H. I am concerned about disadvantages at work related to pregnancy.
    I. I am worried about losing my job or being reassigned due to pregnancy.
    J. My colleagues feel burdened by my pregnancy.
    K. My work performance has decreased since pregnancy.
    L. Taking parental leave is difficult.
    M. I am worried that my working conditions may harm my baby's health.
39. **What is your highest level of education completed?**
40. **What is your average monthly income?**
41. **What is your spouse's average monthly income?**
42. **Over the past two weeks, how often have you been bothered by the following problems?**
    A. Little interest or pleasure in doing things
    B. Feeling down, depressed, or hopeless
    C. Trouble falling or staying asleep, or sleeping too much
    D. Feeling tired or having little energy
    E. Poor appetite or overeating
    F. Feeling bad about yourself — or that you are a failure or have let yourself or your family down
    G. Trouble concentrating on things, such as reading the newspaper or watching television
    H. Moving or speaking so slowly that other people could have noticed, or being so restless that you have been moving around a lot more than usual
    I. Thoughts that you would be better off dead or of hurting yourself in some way
    J. **If you answered "yes" to any of the above, how difficult have these problems made it for you to work, take care of things at home, or get along with other people?**
43. I have been able to laugh and see the funny side of things.
44. I have looked forward with enjoyment to things.
45. I have blamed myself unnecessarily when things went wrong.
46. I have felt anxious or worried without a good reason.
47. I have felt scared or panicky for no good reason.
48. Things have been getting on top of me.
49. I have been so unhappy that I have had difficulty sleeping.
50. I have felt sad or miserable.
51. I have cried because I felt unhappy.
52. I have had thoughts of harming myself.
53. **I feel restless and find it difficult to sit still.**
    A. Feeling nervous, anxious, or on edge
    B. Not being able to stop or control worrying
    C. Trouble relaxing
    D. Being so restless that it is hard to sit still

E. Becoming easily annoyed or irritable
F. Feeling afraid as if something awful might happen

54. **I am satisfied with my current marital relationship.**
55. **How are household duties currently divided between you and your spouse?**
    Self: ___% Spouse: ___%
56. **Do you currently have children you are raising?**
57. **How do you expect childcare responsibilities to be divided between you and your spouse in the future?**
    Self: ___% Spouse: ___%
58. **How are childcare responsibilities currently divided between you and your spouse?**
    Self: ___% Spouse: ___%
59. **(If pregnant) How do you expect childcare responsibilities to be divided after childbirth?**
    Self: ___% Spouse: ___%
60. **To what extent can you receive help with household chores or childcare from people other than your spouse?**
61. **Please indicate the response that best reflects your thoughts about your spouse.**
    A. My spouse shows interest in me.
    B. My spouse understands how I feel.
    C. My spouse recognizes my true value.
    D. When I have serious problems, I rely on my spouse.
    E. When I have concerns, I talk to my spouse about them.
    F. I feel comfortable when I am with my spouse.
62. **Please read each statement and select the option that best describes your current situation.**
    A. There is a special person who is around when I am in need.
    B. There is a special person with whom I can share my joys and sorrows.
    C. My family really tries to help me.
    D. I get the emotional help and support I need from my family.
    E. There is a special person who is a real source of comfort to me.
    F. My friends really try to help me.
    G. I can count on my friends when things go wrong.
    H. I can talk about my problems with my family.
    I. I have friends with whom I can share my joys and sorrows.
    J. There is a special person in my life who cares about my feelings.
    K. My family is willing to help me make decisions.
    L. I can talk about my problems with my friends.
63. **Please indicate the response that best reflects your thoughts about having a child.**

    A. Having a child is worth making any sacrifice.
    B. Becoming a parent has brought me great joy and pleasure.
    C. A close relationship with my child is a special source of joy for me.
    D. Objectively, I am generally happy as a parent.
    E. Watching my child grow and develop is truly enjoyable.
    F. Life as a parent can be considered one of the most important parts of life.
    G. I consider raising a child to be one of the main goals in my life.
    H. I do not find being (or becoming) a parent particularly enjoyable.

I. I gain great satisfaction from being able to provide my child with a good home.
J. Having and raising a child is one of the most exciting things I can imagine.
K. Because I really enjoy (or look forward to) being with my child, I try (or will try) to spend as much time with my child as possible.
L. A child is a major burden to me.
M. Being a parent is always enjoyable.
N. Even thinking about the time I spent (or will spend) with my child makes me happy.
O. I really like talking about my child (or fetus).
P. Having a child to care for is truly enjoyable.
Q. I enjoy spending time looking at (or imagining) my child.

64. During pregnancy or after childbirth, what new physical changes have you experienced, and to what extent do they currently cause discomfort?

A. Fatigue
B. Dizziness
C. Weight gain
D. Hair loss
E. Edema (swelling)
F. Skin pigmentation change – linea nigra (pregnancy line)
G. Skin pigmentation change – axilla (underarm)
H. Skin pigmentation change – groin
I. Skin change – stretch marks
J. Skin change – itching
K. Skin change – other
L. Morning sickness – vomiting
M. Morning sickness – nausea
N. Morning sickness – indigestion
O. Constipation
P. Hemorrhoids
Q. Increased appetite
R. Decreased appetite
S. Bleeding
T. Increased vaginal discharge
U. Perineal itching
V. Pain – lower abdomen
W. Pain – headache
X. Pain – joint pain
Y. Pain – back pain
Z. Pain – breast pain
AA. Pain – other body area(s)
BB. Urinary frequency
CC. Nocturia
DD. Urinary incontinence
EE. Increase in breast size
FF. Insomnia
GG. Depressed mood

HH. Anxiety

65. The following items ask about how you think about yourself. Please read each statement and mark the option that best reflects your thoughts.

A. Pregnancy makes me feel less attractive.
B. I worry that my spouse/partner may find me unattractive during pregnancy.
C. I worry that pregnancy may affect the shape of my breasts.
D. I compare my body negatively with other pregnant women.
E. I feel that I am gaining weight, or that I have gained too much weight.
F. I worry about losing weight after childbirth.
G. I am worried about stretch marks.
H. I worry about how my body will look after childbirth.
I. I worry that my spouse/partner may find me unattractive after childbirth.
J. I worry about how the shape of my breasts will look after childbirth.
K. I have dieted during pregnancy to avoid gaining too much weight.
L. I have tried to limit weight gain during pregnancy.
M. Comments from others about my pregnant body make me feel embarrassed.

66. Please read the following statements and mark the option that best reflects your thoughts.

A. I feel burdened because I expect abnormal results in prenatal screening/tests.
B. I worry that the fetus may have abnormalities.
C. I worry whether the fetus will grow well.
D. I worry about having a miscarriage.
E. I worry about developing pregnancy-related complications.
F. I worry about having a preterm birth.
G. Attending prenatal education classes feels burdensome.
H. Becoming a mother feels burdensome.
I. I find it difficult to calm my emotions for the baby.
J. I worry about emotionally interacting/bonding with the baby.
K. Preparing for the maternal role feels burdensome.
L. Preparing to take care of the baby feels burdensome.
M. If there is a problem with our baby, society seems to believe the mother is the main cause.
N. Society seems to assume that the primary responsibility for childcare rests with the mother.
O. Society seems to expect that mothers will have difficulty continuing their own lives after childbirth.
P. I am concerned that there are insufficient trustworthy childcare facilities in our society.
Q. Our society has high expectations of what a “good mother” should be.

67. If you had anxiety related to pregnancy or childbirth, were there ways to address/resolve it?

68. If yes, what strategies or approaches did you use to address it?

69. If no, what specific issues made it difficult to find solutions or obtain support?

70. What type of solution or support do you think would be most helpful?

71. What do you think about developing a non-pharmacological digital therapeutic for maternal depression/anxiety?

72. What is the reason for your answer?

73. The perinatal mental health digital therapeutic app under development will include a self-assessment function for depressive symptoms to support self-management of mental health, and will provide app-based interventions. If in-person treatment is needed based on the self-assessment results, the app will also provide guidance.

74. The self-assessment data will be stored, and clinicians will be able to review the patient's data during in-person visits.

75. What additional components do you think are needed?

76. A partner/spouse version of the perinatal mental health digital therapeutic app is also being planned. It will provide information on the physical and psychological changes experienced during pregnancy, assess the mental health status of the mother and the couple, and provide guidance on self-management strategies and/or the need for in-person treatment. What do you think about this service?

77. If the perinatal mental health digital therapeutic app provides the following information, how necessary do you think it would be?

A. Medical information about perinatal depression and anxiety
B. Medical information about perinatal health management
C. General medical information about pregnancy and childbirth
D. Strategies to improve sleep quality during pregnancy
E. Information on simple home-based exercises (home training)
F. A function to record and review mood changes

78. If you manage your mental health using the perinatal mental health digital therapeutic app, how necessary do you think the following functions would be? *(Rate 1 to 5)*

A. A function to write a daily mood diary
B. A function to record and review meal-based dietary logs
C. A function to measure physical activity level
D. A function to record and review exercise
E. A function to record and review stress
F. A function to record and review sleep

79. The following additional functions may be included in the perinatal mental health digital therapeutic app. Please indicate how necessary you think each would be. *(Rate 1 to 5)*

A. A function to communicate with your treating physician
B. A function allowing a coach (e.g., psychotherapist or nurse) to review your data and provide education and counseling
C. A social networking–type function to share experiences and communicate with patients in similar situations
D. A function to access the app user manual and instructions at any time
E. A function to ask questions and receive answers from experts
F. A function to search for answers to questions
G. A function to contact someone at any time in urgent situations and be connected to a hospital
H. A function to personalize the app based on your personal information and health status
I. A function to provide tailored advice based on the information you enter and your current condition
J. A function to share your data with a spouse/partner or other family members (with prior consent/settings)

K. A function to engage in treatment together with a spouse/partner or other family members (with prior consent/settings)
L. Reminder function (e.g., reminders for blood glucose checks, mealtimes, etc.)
M. A function to remind you of hospital appointment times
N. A reward function for active health management (e.g., earning points that can be redeemed for rewards)

80. Please freely describe any additional functions or content you think are needed in the perinatal mental health digital therapeutic app beyond the questions above.

81. If an obstetrician/gynecologist prescribes a perinatal mental health digital therapeutic to improve your mental health, would you be willing to use it?
