# Supplementary material for: Factors Associated with Perinatal Depression and Anxiety Among Pregnant and Postpartum Women: A Cross-Sectional Study Based on Questionnaire Data
Source: Diseases. 2026 Feb 11;14(2):67. doi: 10.3390/diseases14020067 (PMC12939594; doi:10.3390/diseases14020067)
Supplement: Supplementary file 1 [file diseases-14-00067-s001.zip › Supplementary Material S4 . cronbach's alpha.pdf]

Supplementary Table S1. Internal Consistency of the Psychometric Instruments

Scale	Number of items	Cronbach's alpha
Patient Health Questionnaire-9 (PHQ-9)	9	0.86
Korean version of the Edinburgh Postnatal Depression Scale (K-EPDS)	10	0.89
Generalized Anxiety Disorder-7 (GAD-7)	7	0.9

Cronbach's alpha coefficients were calculated to assess the internal consistency of each scale in the current study sample. Values ≥ 0.70 indicate acceptable internal consistency.
